# Ontology-Based Classification and Analysis of Adverse Events Associated With the Usage of Chloroquine and Hydroxychloroquine

**DOI:** 10.3389/fphar.2022.812338

**Published:** 2022-03-23

**Authors:** Jamie Ngai, Madison Kalter, James Brian Byrd, Rebecca Racz, Yongqun He

**Affiliations:** ^1^ College of Pharmacy, University of Michigan, Ann Arbor, MI, United States; ^2^ College of Literature, Science, and Arts, University of Michigan, Ann Arbor, MI, United States; ^3^ Department of Internal Medicine, Division of Cardiovascular Medicine, University of Michigan Medical School, Ann Arbor, MI, United States; ^4^ Division of Applied Regulatory Science, Center for Drug Evaluation and Research, US Food and Drug Administration, Silver Spring, MD, United States; ^5^ Unit for Laboratory Animal Medicine, University of Michigan Medical School, Ann Arbor, MI, United States; ^6^ Department of Microbiology and Immunology, University of Michigan Medical School, Ann Arbor, MI, United States; ^7^ Center for Computational Medicine and Bioinformatics, University of Michigan Medical School, Ann Arbor, MI, United States

**Keywords:** chloroquine, hydroxychloroquine, COVID-19, drug, adverse event, ontology, FAERS database

## Abstract

Multiple methodologies have been developed to identify and predict adverse events (AEs); however, many of these methods do not consider how patient population characteristics, such as diseases, age, and gender, affect AEs seen. In this study, we evaluated the utility of collecting and analyzing AE data related to hydroxychloroquine (HCQ) and chloroquine (CQ) from US Prescribing Information (USPIs, also called drug product labels or package inserts), the FDA Adverse Event Reporting System (FAERS), and peer-reviewed literature from PubMed/EMBASE, followed by AE classification and modeling using the Ontology of Adverse Events (OAE). Our USPI analysis showed that CQ and HCQ AE profiles were similar, although HCQ was reported to be associated with fewer types of cardiovascular, nervous system, and musculoskeletal AEs. According to EMBASE literature mining, CQ and HCQ were associated with QT prolongation (primarily when treating COVID-19), heart arrhythmias, development of Torsade des Pointes, and retinopathy (primarily when treating lupus). The FAERS data was analyzed by proportional ratio reporting, Chi-square test, and minimal case number filtering, followed by OAE classification. HCQ was associated with 63 significant AEs (including 21 cardiovascular AEs) for COVID-19 patients and 120 significant AEs (including 12 cardiovascular AEs) for lupus patients, supporting the hypothesis that the disease being treated affects the type and number of certain CQ/HCQ AEs that are manifested. Using an HCQ AE patient example reported in the literature, we also ontologically modeled how an AE occurs and what factors (e.g., age, biological sex, and medical history) are involved in the AE formation. The methodology developed in this study can be used for other drugs and indications to better identify patient populations that are particularly vulnerable to AEs.

## Introduction

Generally, adverse events (AEs) are an undesirable experience associated with the use of a medical product, and can or cannot be causally related (see 21 CFR 314.80) (FDA, 2016). AEs can range from mild effects such as abdominal discomfort to more severe effects such as cardiac arrhythmias or acute neurological disorders. AEs may be detected during research or clinical studies or *via* postmarket surveillance. AEs can differ between patient populations, including patients taking the same drug for different indications (Yu et al., 2019). These differences can often be difficult to elucidate and may be overlooked in both traditional and newer pharmacovigilance methods.

To better study various AEs under different conditions, we can rely on the help from biomedical ontologies. A biomedical ontology is a structured vocabulary of computer- and human-interpretable terms and relations among these terms that represent entities in the biomedical world and how they relate to each other. Ontologies play a critical role in data science to facilitate biomedical data normalization, integration, processing, and analyses (Schulz et al., 2013; Hoehndorf et al., 2015). The Ontology of Adverse Events (OAE) is a community-based formal AE ontology designed to standardize and classify different types of AEs arising subsequent to medical interventions. In addition, OAE addresses AE properties and associated factors and supports computer-assisted reasoning (He et al., 2014). The OAE has been used to support drug and vaccine AE data analysis and could be used to identify AE differences between populations (Sarntivijai et al., 2012; He et al., 2014; Xie et al., 2016a; Xie et al., 2016b; Sarntivijai et al., 2016); additionally, OAE has demonstrated better performance in classifying AEs compared to the Medical Dictionary for Regulatory Activities [MedDRA, the default system for standardizing terms of AEs after medical interventions (Brown et al., 1999)] (Sarntivijai et al., 2012; Xie et al., 2016a; Xie et al., 2016b).

With the coronavirus disease 2019 (COVID-19) pandemic spreading worldwide, many FDA-approved drugs are being evaluated for their efficacy in COVID-19 treatment (Sultana et al., 2020). One class of drugs that has been evaluated is the quinoline antimalarials (Foley and Tilley, 1997), which include the FDA-approved drugs chloroquine (CQ) and hydroxychloroquine (HCQ). This drug class was hypothesized to treat COVID-19 based on findings in a related virus, SARS-CoV (Keyaerts et al., 2004). In late March 2020, the FDA issued an Emergency Use Authorization (EUA) for CQ and HCQ to treat certain patients with COVID-19 in a hospital setting (FDA, 2020b). However, on June 15, 2020, the FDA revoked the emergency use authorization of HCQ and CQ for treatment of COVID-19 patients due to lack of efficacy and a significant risk of AEs, including cardiotoxicity (FDA, 2020a). While CQ and HCQ were previously associated with cardiotoxicity, the prominent appearance of this AE in COVID-19 patients raises the possibility that AE profiles associated with these drugs depend on the disease being treated.

The aim of this paper is to develop a new ontology-based approach to evaluate different patient populations for AEs, using the various indications of CQ and HCQ as a use case. While the consensus is that CQ and HCQ are not effective for COVID-19 (Group et al., 2020), many clinical trials have been conducted, providing a significant amount of AE and other clinical data. In this study, we collected and analyzed AE reports from FAERS, literature, and product labels across and within the various indications and model the results in the OAE to identify similarities and differences in AEs. We also tested our hypothesis that CQ and HCQ would have different AE profiles from each other, and that these profiles would differ depending on the indication. The methodology developed in this study is scalable and can be used to identify similarities and differences in AEs between other drugs and their respective indications.

## Methods

### General Workflow of Our Methodology

**FIGURE 1:**
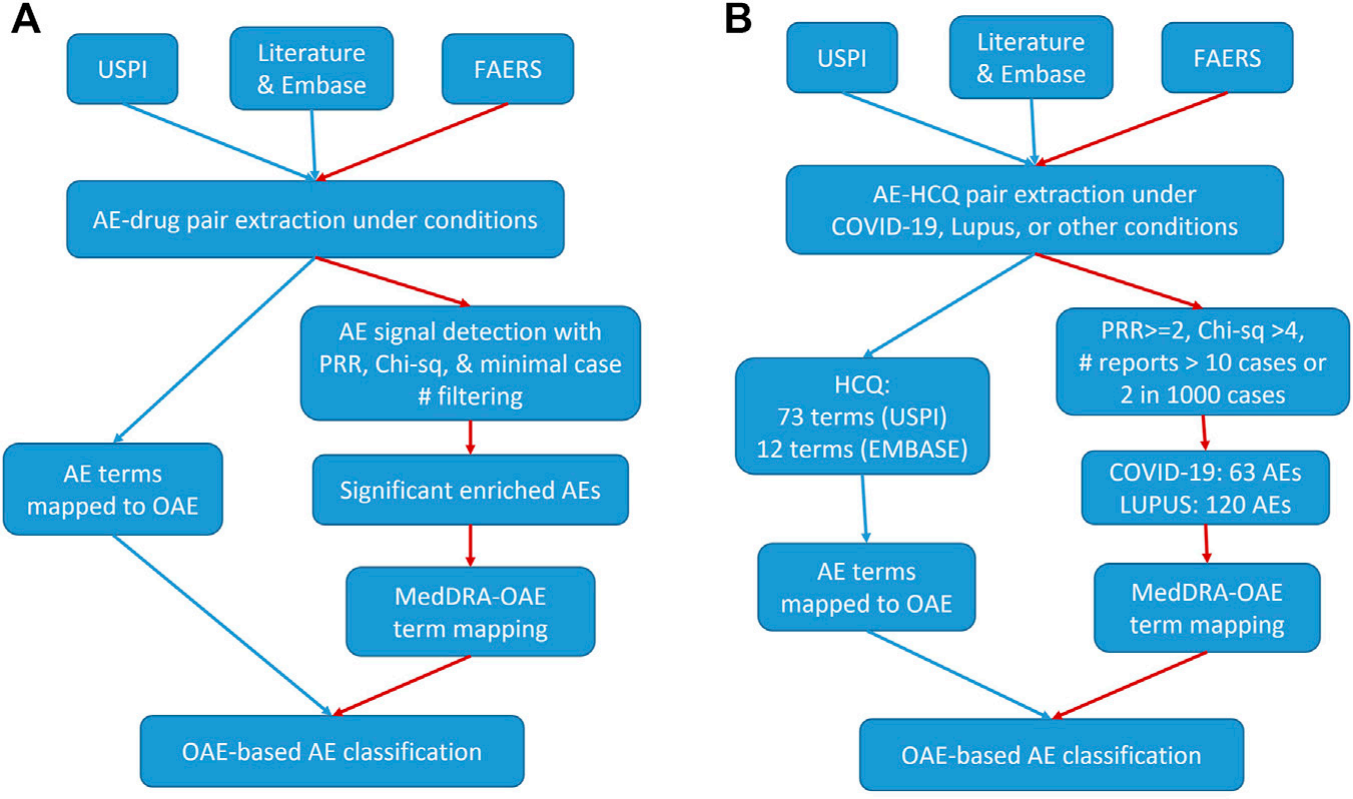
Workflow for our drug AE analysis. **(A)** General workflow. **(B)** HCQ specific workflow. The red paths presented with red edges represent the workflow of FAERS-specific drug AE analyses. USPI, United States Prescribing Information. FAERS, FDA Adverse Event Reporting System. AE, Adverse Event. PRR, Proportional Reporting Ratio. OAE, Ontology of Adverse Event. HCQ, hydroxychloroquine.

Figure 1 shows the workflow of our methodology, with Figure 1A providing the general workflow and Figure 1B providing the workflow for this evaluation of HCQ and CQ. Specifically, our research used three data sources: 1) the United States Prescribing Information (USPI), also known as package insert information, 2) literature databases including PubMed (Lu, 2011) and EMBASE (Wong et al., 2006), and 3) FAERS data. All data were processed and analyzed to extract drugs and their associated AEs under specific conditions (such as COVID-19 infection). Then the AE terms for one or more specific drugs were mapped to the OAE to generate OAE-based AE classifications. Alternatively, for the FAERS data, specific statistical tests, including proportional reporting ratio (PRR) (Evans et al., 2001), Chi-square, and minimal case number filtering tests were performed, and statistically significantly enriched AEs were identified. PRR is often used to measure the extent to which a particular AE is reported for individuals taking a specific drug, compared to the frequency at which the same AE is reported for patients taking other drugs in an AE case report system such as FAERS. These AEs under FAERS were tagged with MedDRA IDs and were then mapped to OAE terms. The OAE-based AE classifications were then used for further classification.

In this study, we used the above-described methodology to analyze the AE differences by indication for HCQ and CQ. The details about the HCQ and CQ AE analysis workflow (Figure 1B) are provided below.

### AE Collection From US Prescribing Information Inserts and Literature

Three drug USPIs, commonly known as a package insert or label, databases were surveyed: Drugs@FDA, DailyMed, and RxDrugLabels, to find AEs associated with name brand and generic quinoline-containing drug products. The FDA USPIs available on September 30, 2020, of the name brand drugs of CQ and HCQ, Aralen^®^ and Plaquenil^®^ respectively, as well as two generic versions of the drugs, were surveyed. AEs listed under the “Adverse Reactions” section were extracted, mapped, and catalogued using the OAE. Ontologies were created to visualize CQ and HCQ’s specific AEs as well as their shared AEs. Meanwhile, the diseases treated by these drugs were also gathered.

AEs reported in published literature as of January 17, 2021, were found through the biomedical bibliographic database, EMBASE (Wong et al., 2006). The usage of CQ/HCQ was at its peak before June 15, 2020 when the EUA was revoked by the FDA due to safety issues and lack of efficacy (FDA, 2020a). The literature reports of their usage dramatically decreased after 2020. Each drug was searched under three disease states: COVID-19, systemic lupus erythematosus (SLE), and rheumatoid arthritis for HCQ, and COVID-19, systemic lupus erythematosus, and malaria for CQ. Results were then filtered by the “Drugs” category to select specific papers using CQ or HCQ. The key subheading, “Adverse drug reaction,” was used to find specific AEs mentioned and provided the number of literature reports where these AEs were reported. The five most commonly reported AEs were collected, along with the number of papers mentioning those AEs and their respective percentages of the total, for each disease state. Results were then analyzed to exclude repeat data.

Additional clinical trials and case reports were found through PubMed using the search terms, “COVID-19”, “chloroquine,” or “hydroxychloroquine,” and “adverse events”, by the date of January 17, 2021. While over 100 clinical trials related to CQ/HCQ exist in clinicaltrials.gov, only five clinical trials were found to have AE results available (as of March 12, 2021).

### AE Collection and Analysis From FAERS

FAERS is a database containing AEs and medication error reports that have been sent to FDA by industry, healthcare providers, patients, and other interested parties. To ensure these data are easily accessible, FDA has developed the FAERS Public Dashboard (https://www.fda.gov/about-fda/update-fda-adverse-event-reporting-system-faers-public-dashboard), a web-based interface that allows for user-friendly querying and organization of FAERS data. The dashboard sorts the data by individual AE or individual product (drug). AEs in the dashboard correspond to MedDRA Preferred Terms, and they are also grouped by “Reaction Group,” which corresponds to the MedDRA System Organ Class (SOC) terms. The AE data for generic CQ, Aralen^®^, generic HCQ and Plaquenil^®^ were downloaded from the dashboard as Excel files, and at the time of the analysis the last quarterly data update had been included on September 30, 2020. Additionally, the total number of reports in FAERS and the number of reports for each AE in FAERS were also recorded.

The Excel spreadsheets contained each AE reported for the individual drug and the number of cases reported for that AE-drug pair. The four products were analyzed separately and then compared. For both HCQ and CQ, the generic drug is more commonly used, and therefore used for further analysis. For HCQ (generic), the data was also sorted by reported indication to compare the incidence of AEs for COVID-19 and systemic lupus erythematosus. HCQ was chosen for further analysis as more cases have been reported in the FAERS database for all indications, which would reduce statistical error. The data was sorted by number of reported AE cases, and any AE with fewer than 10 reported cases or representing less than 0.2% of the number of case reports for that drug was deemed insignificant. Using the data in the spreadsheet, a Chi-square test and PRR test (Evans et al., 2001) were performed for each AE. PRR compares the individual case numbers for each AE to the overall cases in the FAERS system and the overall cases reported for the specific drug. Cases with a Chi-square result of less than 4 (corresponding with a *p*-value > 0.05) and a PRR result of less than 2 were determined to be insignificant (Sarntivijai et al., 2012). The resulting AEs were then sorted back into the high-level Reaction Groups/MedDRA SOCs within Excel, and the number of unique cases for each group was recorded. This was then divided by the total significant AE instances and converted to a percentage to allow for comparison. The resulting lists for each drug were input into the OAE.

### OAE Ontology Term Mapping, Extraction, and Visualization

An online biomedical ontology tool OntoFox (Xiang et al., 2010) was used to extract a subset of the Ontology of Adverse Event that includes the enriched AE terms identified in our data analysis as well as other high level AE terms related to these enriched AE terms. The OntoFox output was then visualized using the Protégè-OWL editor (Musen, 2015). This process develops a visual representation of all the AEs for a given drug and indication, and how they all relate to one another by placing them in groups. For example, “myopathy” is categorized under “muscle AE,” which can then be categorized under the more general “musculoskeletal and connective tissue AE.” This provides a way to directly compare specific types of AEs between different drugs and indications.

## Results

### Comparison of CQ and HCQ AEs From USPIs

**FIGURE 2:**
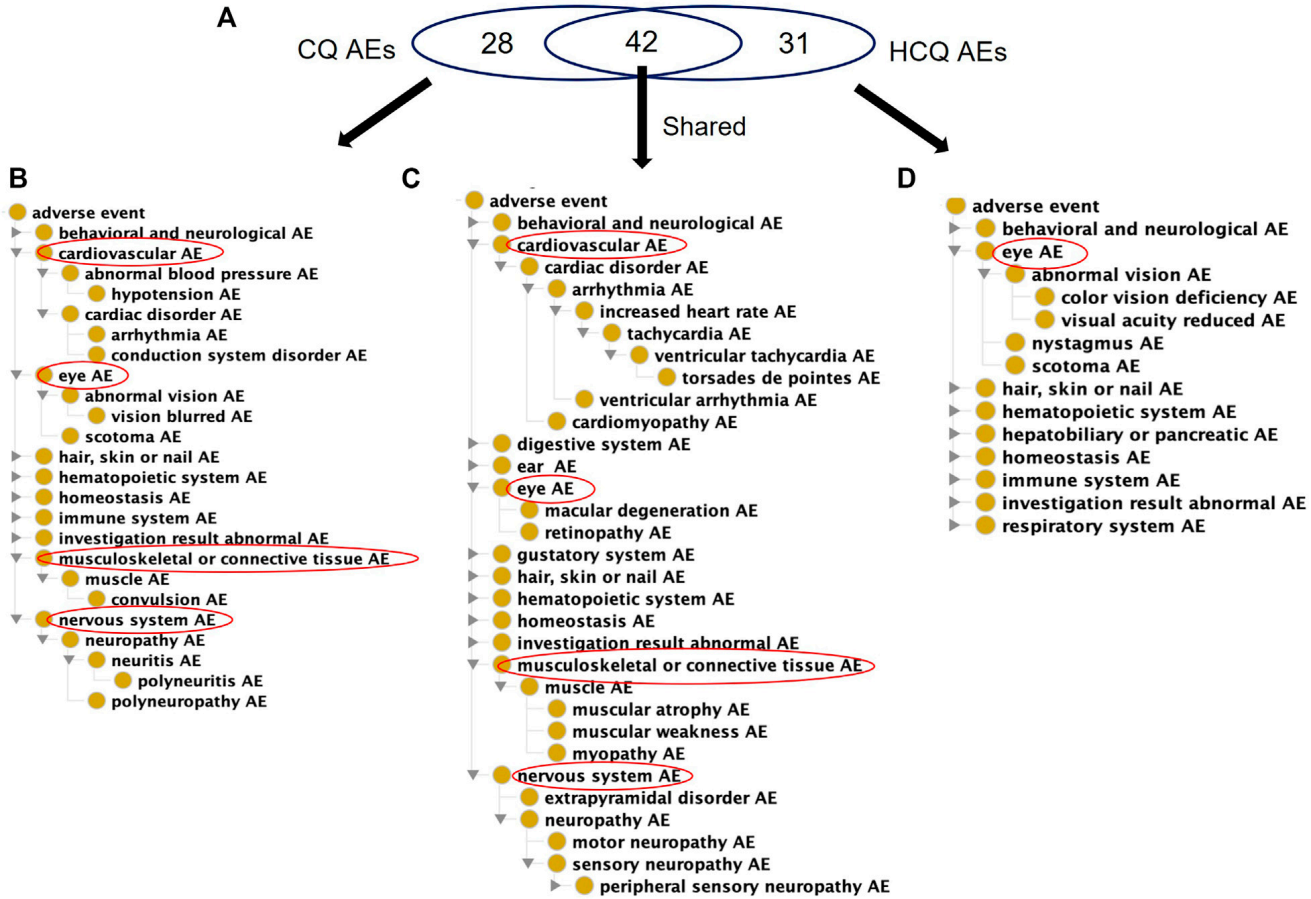
Comparison of USPI-listed AEs associated with chloroquine and Hydroxychloroquine using the OAE. **(A)** Venn Diagram of CQ and HCQ AEs. **(B)** OAE display of 28 CQ-specific AEs **(C)** OAE display of 42 AEs shared by CQ and HCQ. **(D)** OAE display of 31 AEs specific to HCQ. These results were identified using the FDA US Prescribing Information (USPI) data. In each panel in **(B,D)**, cardiovascular and eye AEs are expanded, if present in the list. Overall CQ and HCQ were associated with similar AE profiles, but HCQ had fewer types of severe cardiovascular, nervous, and musculoskeletal AEs.

CQ and HCQ are generally considered safe molecules with low incidences of AEs (James et al., 2007). With their similar structures and both being 4-aminoquinolines, CQ and HCQ have similar AE profiles, with many AEs in common (Figure 2). There is evidence that HCQ, a derivative of CQ, is associated with fewer AEs, although no mechanism has been proposed (Felson et al., 1990; Ruiz-Irastorza et al., 2010). However, CQ has fewer AEs listed on the USPIs compared to HCQ. Although HCQ has more labeled AEs, it lacks specific cardiovascular, nervous system, and musculoskeletal/connective tissue AEs compared to CQ.

### COVID-19 CQ/HCQ AE Profiles From Literature Mined Reports Using EMBASE

AEs from EMBASE, using the filters, “Drugs,” and “Adverse drug reactions,” were used to gather the number of published articles that report the specified AE for both CQ and HCQ, as summarized in Table 1 and Table 2, respectively. The inclusion criteria for the papers screened were to be reporting results from a human randomized clinical trial and published during 2017–2021. The literature regarding COVID-19 were all published during 2019–2021, but because CQ and HCQ possess a long history in both autoimmune and infectious diseases, the inclusion criteria for literature regarding those diseases (rheumatoid arthritis, systemic lupus erythematosus, and malaria) was extended two additional years, back to 2017. This is to ensure the data is current and follows modern AE collection guidelines.

**TABLE 1:** Five AEs most frequently discussed in papers describing chloroquine (CQ) use in various disease states (as discerned by a search of EMBASE).

COVID-19		Systematic Lupus Erythematosus (SLE)		Malaria	
Adverse Event	Frequency (%^a^)	Adverse Event	Frequency (%^a^)	Adverse Event	Frequency (%^a^)
QT Prolongation	38 (43.7%)	Retinopathy	8 (34.8%)	General Side Effects	8 (32%)
Heart Arrhythmia	13 (14.9%)	Cardiomyopathy	6 (26.1%)	Headache	7 (28%)
Torsade des Pointes	13 (14.9%)	Eye Toxicity	5 (21.7%)	Retinopathy	7 (28%)
Retinopathy	11 (12.6%)	Heart Arrhythmia	4 (17.4%)	QT Prolongation	6 (24%)
General Side Effects	9 (10.3%)	Diarrhea	3 (13.0%)	Nausea	5 (20%)

a Percentage of papers reporting this AE, as classified by the EMBASE database.

**TABLE 2:** Five AEs most frequently discussed in papers describing hydroxychloroquine (HCQ) use in various disease states (as discerned by a search of EMBASE).

COVID-19		Systemic lupus erythematosus (SLE)		Rheumatoid arthritis (RA)	
Adverse Event	Frequency (%^a^)	Adverse Event	Frequency (%^a^)	Adverse Event	Frequency (%^a^)
QT Prolongation	133 (43.9%)	Retinopathy	39 (26.7%)	Retinopathy	23 (17.3%)
Diarrhea	47 (15.5%)	Cardiomyopathy	13 (8.9%)	Rash	13 (9.8%)
Nausea	47 (15.5%)	Eye Toxicity	12 (8.2%)	Infection	12 (9.0%)
Heart Arrhythmia	37 (12.2%)	Rash	12 (8.2%)	Nausea	12 (9.0%)
Torsade des Pointes	35 (11.6%)	Diarrhea	11 (7.5%)	General Side Effect	11 (8.3%)

a Percentage of papers reporting this AE, as classified by the EMBASE database.

Of 87 published papers that were reviewed for CQ use for COVID-19, 38 (43.7%) reported the development of QT prolongation, 13 (14.9%) reported the development of heart arrhythmias, and 13 (14.9%) reported the development of Torsade des Pointes. Only 11 (12.6%) reported retinopathy, an AE commonly associated with CQ use. 23 published articles on CQ use for SLE were reviewed, of which 6 (26.1%) reported cardiomyopathy as an AE. The most prevalent AE associated with CQ use for SLE are eye AEs, including retinopathy (34.8%) and eye toxicity (21.7%). Additionally, 25 articles on CQ use for malaria were reviewed, of which the most common AEs include general side effects, headache, and retinopathy. QT prolongation was reported only in 6 (24%) articles.

This trend holds true when observing HCQ use for COVID-19 and SLE. QT prolongation is also the most prevalent AE reported in the 303 published articles on HCQ use for COVID-19, with other cardiac AEs being heart arrhythmias and Torsade des Pointes. HCQ use for SLE (lupus) resulted in similar AEs as CQ use for SLE, with cardiomyopathy being reported in 8.9% of the 146 published articles compared to retinopathy (26.7%) and eye toxicity (8.2%). As for the 133 published articles on HCQ use for RA, retinopathy was the most reported AE, followed by rash, infection, nausea, and general side effects.

### COVID-19 CQ/HCQ AE Profiles From Clinical Trial and Observational Studies Reports

AEs associated with COVID patients treated with CQ and HCQ were collected. At the time of reviewing the related works, not many studies have been conducted. Although many reports focus on HCQ, only two studies focused on CQ. Therefore, these two studies were surveyed for CQ. Three representative HCQ studies were also surveyed. The results are summarized in Table 3 and described below.

**TABLE 3:** AEs associated with CQ/HCQ administration were reported in two clinical trials and three observational studies where COVID-19 patients were given either CQ or HCQ in combination with standard of care drugs.

Drug/Location/References	Clinical Trial No./PubMed No	AEs Reported
Chloroquine (Rosenberg et al., 2020)	PMID: 32236562	Vomiting; abdominal pain; nausea; diarrhea; rash/itchiness; cough; shortness of breath
Chloroquine (Borba et al., 2020)	NCT04323527 PMID: 32330277	Decreased hemoglobin; increased creatinine; increased CK; increased CKMB; QTcF >500 m; ventricular tachycardia
Hydroxychloroquine (Tang et al., 2020)	PMID: 32409561	Diarrhea; vomiting; nausea; abdominal discomfort; thirst; sinus bradycardia; hypertension; orthostatic hypotension; hypertriglyceridemia; decreased appetite; fatigue; dyspnoea; flush; kidney injury; coagulation dysfunction; blurred vision; decreased WBC; increased alanine aminotransferase; increased serum amylase; decreased neutrophil count; disease progression*; upper respiratory tract*
Hydroxychloroquine (Rosenberg et al., 2020)	PMID: 32392282	Diarrhea; hypoglycemia; cardiac arrest*; abnormal ECG*; arrhythmia*; QT prolongation*
Hydroxychloroquine (RECOVERY Group, (Group et al., 2020)	NCT04381936	Atrial flutter/fibrillation, other supraventricular tachycardia, ventricular tachycardia, ventricular fibrillation, atrioventricular block requiring intervention

Serious AEs, as defined by the respective study investigators, are marked with *.

In a 2018 study in China, no serious AEs were reported (Huang et al., 2020). Out of ten COVID-19 patients treated with CQ, five reported AEs. The most frequent AEs were vomiting (50%), diarrhea (50%), nausea (40%), and cough (40%). Other non-serious AEs such as abdominal pain, rash, and shortness of breath were also reported (10%) (Huang et al., 2020). However, in a 2020 randomized clinical trial held in Brazil, high and low dosages of CQ were compared in patients with severe COVID-19 (Borba et al., 2020). The lower dose group (450 mg daily) showed fewer cases of AEs including decreased hemoglobin, increased creatinine, increased creatine kinase, and increased CK-MB, an isoenzyme of creatine kinase. Seven of thirty-seven patients (19%) receiving the high dosage reported a prolonged QTc interval compared to one patient receiving the low dosage, and two patients receiving the high dosage reported symptoms of ventricular tachycardia compared to zero patients receiving the low dosage (Borba et al., 2020).

In a 2020 study conducted in China, seventy-five COVID-19 patients were treated with HCQ with 30% of them reporting AEs. Three percent of patients reported serious AEs, including disease progression (1%) and upper respiratory tract infection (1%). The other 27% of patients who reported AEs reported non-serious AEs, the most common ones being diarrhea (10%) and vomiting (3%). Other AEs commonly associated with HCQ such as blurred vision, fatigue, and abdominal discomfort were only reported once each (Tang et al., 2020). In the second HCQ trial in New York (2020) involving 1438 participants, diarrhea was reported in 17% of patients treated with HCQ alone and 11.6% of patients treated with HCQ in conjunction with azithromycin, a broad-spectrum antibiotic used in treating infections present in COVID-19 patients. The New York study also reported serious cardiac AEs such as cardiac arrest (13.7%), abnormal ECG (27.3%), arrhythmia (16.2%) and QT prolongation (14.4%) in COVID-19 patients treated with HCQ alone (Rosenberg et al., 2020). In the impactful study done by the RECOVERY Group, who treated 1,561 COVID-19 patients with HCQ, 60 (8.2%) developed major cardiac arrhythmias compared to 90 of 3,155 (6.3%) of patients who received the usual care. The most common cardiac AEs reported were supraventricular tachycardias, with 56 (7.6%) patients from the HCQ group and 74 (5.2%) patients in the usual care group (Borba et al., 2020).

### Differential FAERS AE Profiles by CQ and HCQ

In total, 1,998 AE case reports were collected for CQ and Aralen^®^ with possibility for duplication. A total of 908 different AEs were reported for CQ and 351 different AEs were reported for Aralen^®^. After statistical analysis (see the Methods section for detail), 78 AEs met or exceeded the significance thresholds for CQ, and 63 AEs met or exceeded the significance thresholds for Aralen^®^. The three most frequent reaction groups among the significant AEs for CQ were Cardiac Disorders, Nervous System Disorders, and Eye Disorders. The two most frequent reaction groups among the significant AEs for Aralen^®^ were Eye Disorders, and Nervous System Disorders. Under the source term there were 15 subheadings for CQ and 16 subheadings for Aralen^®^.

In total, 36,641 AE reports were collected for HCQ and Plaquenil^®^. A total of 3,486 different AEs were reported for HCQ, and 2,796 different AEs for Plaquenil^®^. After statistical data analysis, 353 AEs met or exceeded the significance thresholds for HCQ and 303 AEs for Plaquenil^®^. The three most frequent reaction groups among the significant AEs for HCQ and Plaquenil^®^, analyzed separately but with the same result, were General Disorders and Administration Site Conditions (top condition: drug ineffective AE), Musculoskeletal and Connective Tissue Disorders (top condition: rheumatoid arthritis AE), and Gastrointestinal Disorders (top condition: nausea AE). Under the top-level source term in OAE there were 23 subheadings for HCQ and 21 subheadings for Plaquenil^®^.

**FIGURE 3:**
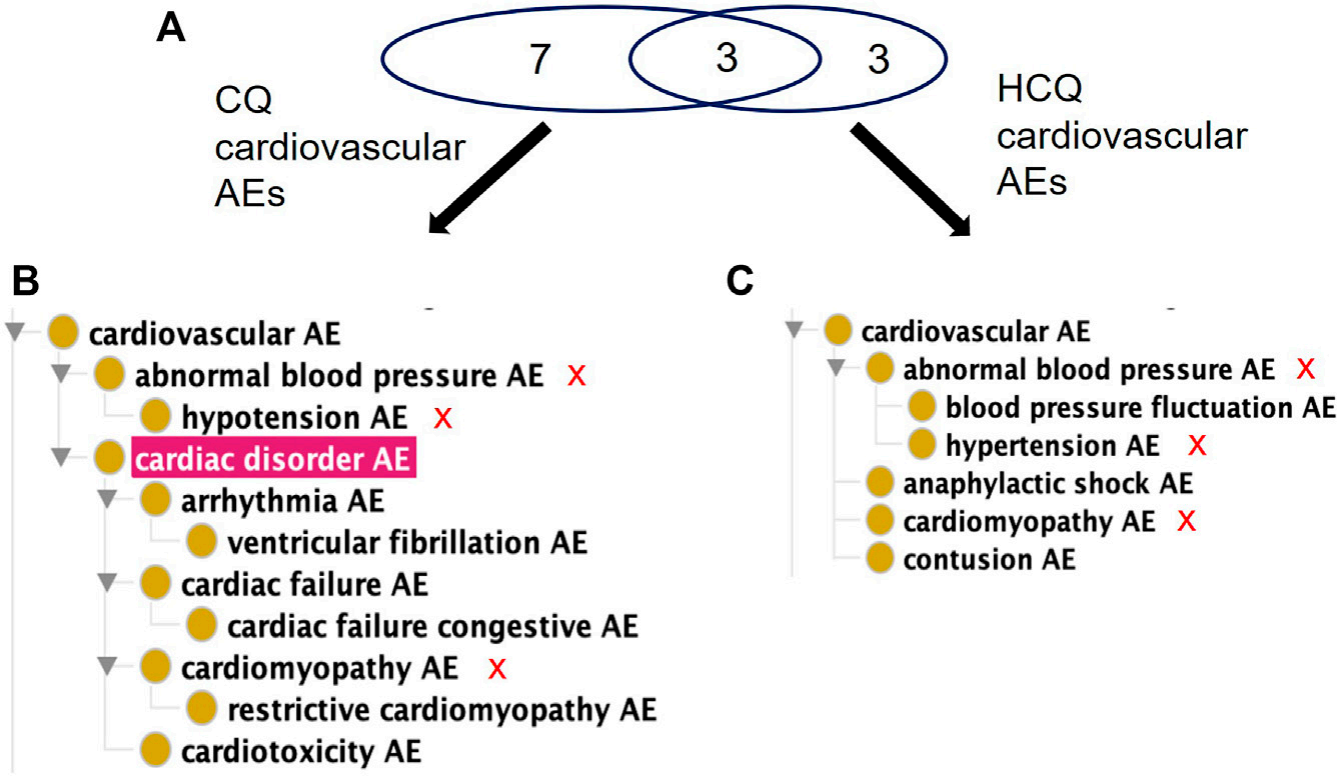
Comparison of significant cardiovascular AEs for CQ and HCQ from the FAERS database. **(A)** Venn Diagram of significant cardiovascular AEs for CQ and HCQ. **(B)** OAE classification of 10 significant cardiovascular AEs for CQ. (C) OAE classification of 6 significant cardiovascular AEs for HCQ. The red sign of “X” next to the AE terms in **(B,C)** represent the shared AE for CQ and HCQ. Note that cardiac disorder AE passed the significance threshold for CQ **(A)** but not for HCQ **(B)**. Overall CQ was associated with more cardiovascular AEs.

When considering FAERS reports across all indications, the percentage of significant AE cases for just CQ that falls in the FAERS reaction group of Cardiac Disorder is about 20.5% compared to 0.32% for HCQ. OAE’s Cardiovascular AE (http://purl.obolibrary.org/obo/OAE_0000493) is highlighted in Figure 2. The number of AEs under the Cardiovascular AE heading for CQ (Figure 3A) is much larger than the number of AEs under the Cardiovascular AE heading for HCQ (Figure 3B). Compared with the results from USPIs (Figure 2), more cardiovascular AE term types were reported in both CQ and HCQ cases in FAERS. This could be a result of increased use after the EUA and stimulated reporting after news of cardiac complications. HCQ reports doubled in the year 2020, 10,362 compared to 5,042.

### Differential FAERS AE Profiles in Systemic Lupus Erythematosus and COVID-19 Patients Treated With HCQ

**FIGURE 4:**
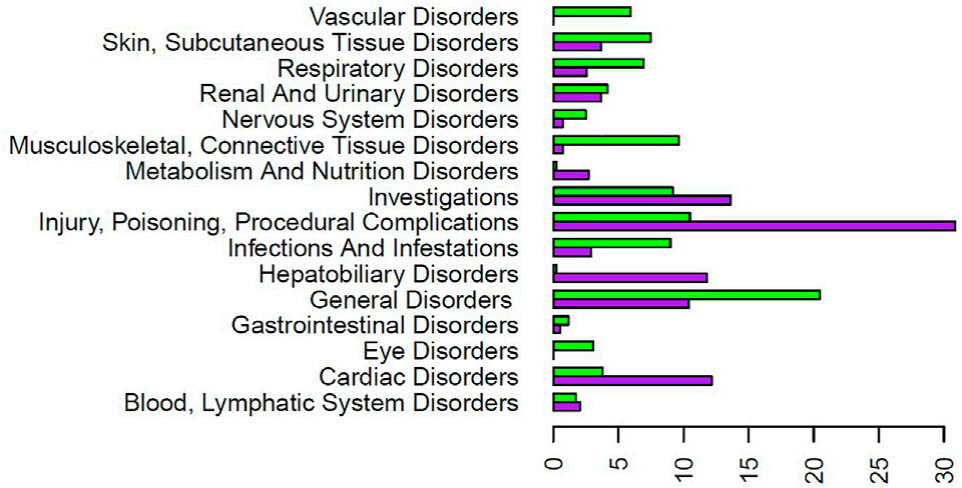
Significant AE reaction group percentage comparison for COVID-19 and SLE patients treated with HCQ from the FAERS database. The purpose bars represent the percentage of the total significant count for COVID-19 for each reaction group. The green bars represent the percentage of the total significant count for SLE for each reaction group. The reaction groups of Ear and Labyrinth Disorders, Immune System Disorders, Pregnancy, Puerperium and Perinatal Conditions, Product Issues, Psychiatric Disorders, Social Circumstances, and Surgical and Medical Procedures were left off the graph due to small percentages for both patient types. Note that only the AEs that have passed the significance test were used here for all calculations). HCQ AE patterns appeared different depending on the diseases treated.

The FAERS database had 1,002 case reports for COVID-19 patients and 1,527 case reports for SLE patients treated with HCQ. After analysis, there were 63 significant AE types for COVID-19 patients and 120 significant AE types for SLE patients (Supplementary Table S1). When broken into MedDRA higher level term percentages, of significant cases for each separate disease, the categories with the greatest differential were Cardiac Disorders, General Disorders and Administration Site Conditions, and Injury, Poisoning and Procedural Complications (Figure 4). Compared to HCQ, a greater percentage of the significant cases for both COVID-19 and SLE were cardiac disorders; however, cardiac disorders are markedly more reported in COVID-19 patients (i.e., 12.2%) than that in SLE patients (3.8%) taking the same drug (Figure 4).

**FIGURE 5:**
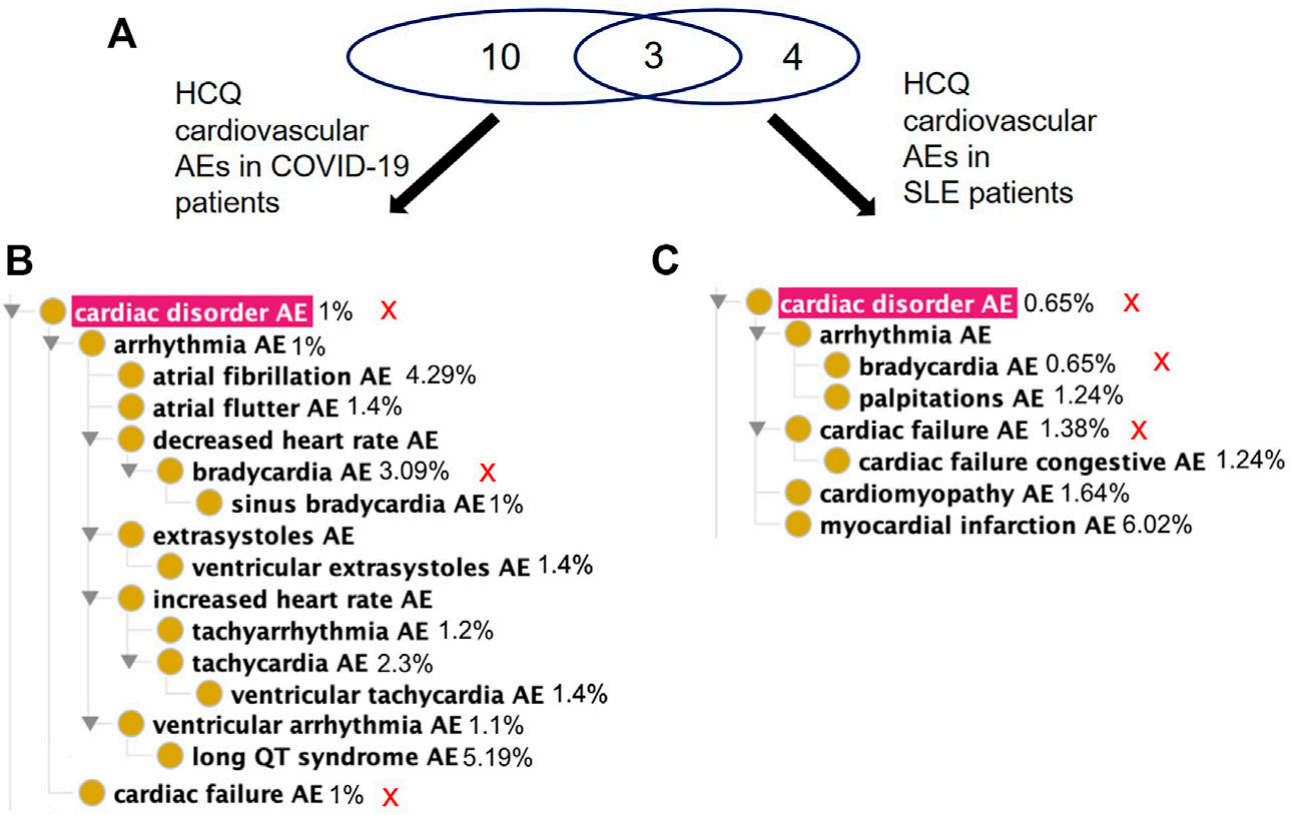
Comparison of significant cardiac AEs for COVID-19 and SLE patients treated with HCQ from the FAERS database. **(A)** Venn diagram of significant cardiac AEs for COVID-19 and SLE patients treated with HCQ. **(B)** OAE classification of significant cardiac AEs for COVID-19 patients treated with HCQ. **(C)** OAE classification of significant cardiac AEs for SLE patients treated with HCQ. The percentages next to the AE terms represent the occurrence rates of these AEs under the specific condition. The red sign of “X” next to the AE terms in **(B,C)** represent the shared AE for the two types of patients. Different cardiac AEs were found to be associated with HCQ following its usage for treating COVID-19 and lupus.

Of the 63 significant AEs for COVID-19 patients treated with HCQ, 21 are classified by OBO as Cardiovascular AE (http://purl.obolibrary.org/obo/OAE_0000493), a concern FDA has previously highlighted in association with the treatment of COVID-19 patients with quinoline drugs (Arshad et al., 2020). Of the 120 significant AEs for SLE patients treated with HCQ, 12 fall under Cardiovascular AE (http://purl.obolibrary.org/obo/OAE_0000493). In addition, 14 AEs fall under the Cardiovascular AE sublevel Cardiac Disorder AE (http://purl.obolibrary.org/obo/OAE_0000084) for COVID-19 patients compared to 7 AEs for SLE patients (Figure 5). Notably, there were more reports of long QT syndrome, atrial fibrillation, and bradycardia in COVID-19 compared to SLE. This demonstrates that AE profiles for a drug may differ between indications.

### Drug AE Condition Modeling and Classification

**FIGURE 6:**
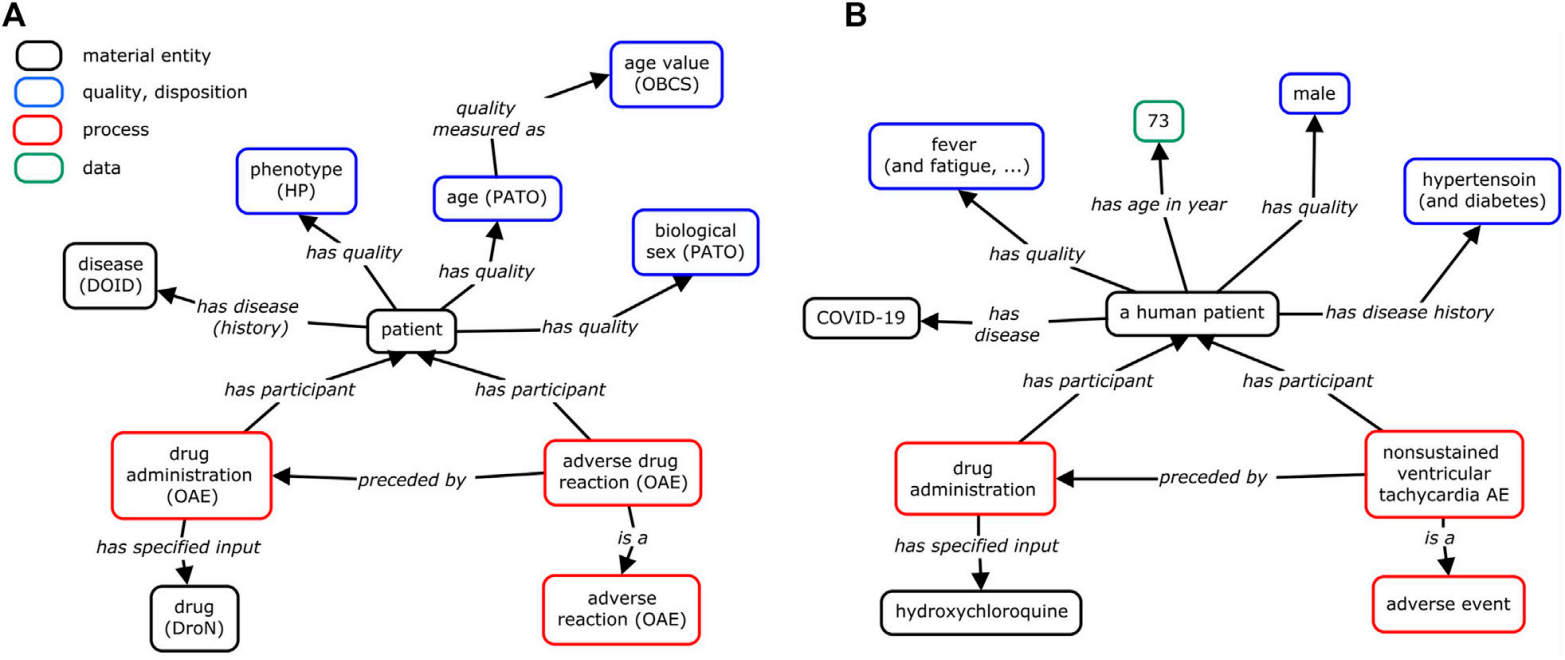
Modeling of condition-dependent AE occurrence with an example published in PubMed. **(A)** General modeling. **(B)** HCQ AE case study example. In this example, a human patient with COVID-19 was treated with HCQ, and later suffered from unsustained ventricular tachycardia AE (Abdelmaseih et al., 2020). In addition to the positive COVID-19 diagnosis, the study of the HCQ AE needs to consider the patient’s age (75 years old), biological sex (male), medical disease history (hypertension and diabetes), and the symptoms (e.g., fever, dry cough) before the usage of the HCQ drug. If these conditions had changed, the cardiac disease might have not occurred. To support ontology interoperability, OAE also reuses terms from other ontologies, including Human Phenotype Ontology (HP) (Kohler et al., 2021), Drug Ontology (DrON) (Hanna et al., 2013), disease Ontology (DOID) (Schriml et al., 2012), Phenotype And Trait Ontology (PATO) (Mabee et al., 2007), and Ontology of Biological and Clinical Statistics (OBCS) (Zheng et al., 2016). The relation “has age (in year)” is a shortcut relation of (“has quality” some (age and “quality measured as (in year)”)). This method of ontology modeling allows us to better visualize the many factors that contribute to the manifestation of certain AEs.

While our study has so far focused on the analysis of the difference between AE manifestation between SLE and COVID-19 patients following CQ/HCQ drug administration, AEs occur under specific conditions that also include many other factors than the disease type. Figure 6A provides a general OAE modeling and classification of how an AE occurs and what factors are involved in the generation of the AE. Basically, an AE of a patient occurs after a drug administration in the patient after its diagnosis of a specific disease with specific disease symptoms or signs. The patient’s qualities (e.g., age and biological sex) and medical history are associated with and may affect the manifestation of the AE.

The general model shown in Figure 6A can be illustrated with specific patient cases. As part of the literature review, many case reports (*n* = 1) have been published as observational reports. We have selected one such case report in which a COVID-19 patient developed cardiac AEs after beginning HCQ treatment (Abdelmaseih et al., 2020). The details of the case were mapped (Figure 6B) to help identify variables that could potentially affect disease state and AE outcomes. This is the modeling of the case study of a 75-year-old male presenting to the emergency room with worsening shortness of breath, dry cough, fatigue, and high fever (Abdelmaseih et al., 2020). The patient had a medical history of hypertension and diabetes. After testing positive for COVID-19, the patient was treated with HCQ and subsequently developed episodes of unsustained ventricular tachycardia, which resolved after termination of HCQ. In this case, while the administration of HCQ in the COVID-19 patient was associated with a cardiac AE, a better understanding of this case requires our consideration of the patient’s specific conditions including age, biological sex, and medical history.

The ontology modeling approach (Figure 6) facilitates the identification of variables that may affect the AE outcomes in COVID-19 or other disease patients given different conditions. In addition to the diseases to be treated, patient qualities such as age and biological sex, preexisting health conditions, other factors such as drug dosage can be included. OAE treats the AE as a pathological bodily process that is a dynamical process and thus may require dynamic monitoring. For example, given that the half-lives of CQ and HCQ are long (approximately 1–2 months), long-term monitoring for their safety profiles is also needed (Gevers et al., 2020). As a result, we could expect the generation of a metadata (i.e., the data that describe the representation of instance data) standard that standardizes these AE-associated variables and their measurement for better case reports and data analysis. Ontology modeling and standardization can provide support to such process.

## Discussion

In this study, we utilized a systematic methodology to analyze and classify AEs across the various indications of two drugs, CQ and HCQ. First, we developed a formal survey and analysis pipeline using three major resources (i.e., USPIs, literature resources including EMBASE and PubMed, and FAERS database) to consistently analyze AEs given difference conditions including the disease being treated. We have emphasized the role of ontology in the AE classification and modeling in our pipeline. Second, we have applied our methodology to study the profiles of CQ and HCQ AEs for treating diseases including COVID-19 and SLE. The findings in this study demonstrated that a systemic methodology leveraging complementary publicly available sources can assist with identifying differences in the number of and type of AEs between different indications of the same drug. For example, the FAERS and EMBASE analyses each demonstrated different AE types given different indications. OAE modeling of these results further supported these conclusions and enabled us to semantically represent different conditions under which an AE may be identified. In summary, although common AEs were found, our results identified many distinct AE results given various conditions, including different diseases treated by these two drugs.

One major focus of this paper was on the methodology for evaluating AEs (Figure 1). While AEs for multiple indications may be labeled on one USPI, there may be differences in AE rates between indications. Therefore, multiple sources were evaluated, including literature, USPIs, and FAERS. Beyond this analysis, ontology modeling facilitated the identification of similarities and differences in AE profiles. Ontologies have emerged to become important for standard data and knowledge representation, classification, and analysis. OAE provides a standard ontology method for classifying and analyzing various AEs. Previous studies demonstrated that OAE performed better than MedDRA (a commonly used AE presentation standard for AE case reporting) in ontology classification analysis (Sarntivijai et al., 2012; He et al., 2014; He et al., 2016; Xie et al., 2016a). In the current study, we used USPI data, FAERS data, and literature data in correlation to compare AE profiles of drugs in the same class and of one drug in different diseases. Our OAE-based AE classification method clearly shows the hierarchical structure of identified AEs and allows us to quickly group various AEs into specific categories. In addition, OAE can be used to model and represent AE formation processes in individual patients as shown in Figure 6, which can be further used to support AE data standardization and analysis (He et al., 2014; Wong et al., 2017).

This methodology demonstrated several strengths for evaluating AEs. First, using multiple sources allows for trend identification, signal strengthening, and can help reduce bias that may be present in one source. In this example, while FAERS may have biased reporting, cardiovascular events were also reported in the literature and labels, supporting the possibility that cardiovascular AEs are more prevalent in COVID-19 than SLE patients taking HCQ. Next, incorporating the information from these sources into OAE allows for easy visualization and analysis. In this example, we were able to identify AE differences between HCQ and CQ as well as SLE and COVID-19 *via* ontology modeling. This methodology can be utilized to evaluate other drug-drug pairs or drug-indication pairs for differences; other drugs have shown similar patterns, such as sirolimus (also known as rapamycin), which displays specific AEs (e.g., acne, stomatitis) that manifest when it is used to treat lymphangioleiomyomatosis, yet different AEs (e.g., anemia, hypertension) that manifest when used in renal transplantation (Yu et al., 2019).

Using the methodology described above, we analyzed different data sources related to CQ and HCQ AEs and made many interesting findings. First, our USPI data analysis found that while CQ and HCQ had similar AE profiles, HCQ lacked many cardiovascular, nervous, and musculoskeletal AEs found in CQ, including hypotension, arrhythmia, convulsion, and polyneuropathy AEs (Figure 2). While USPI results came from well-controlled randomized studies, the information about study size were limited in the USPI results. To complement the USPI reports, the EMBASE database includes a large number of studies and results, and the number of EMBASE papers citing specific AEs provides us a feasible way to rank the frequency of AE occurrences. EMBASE data mining found that CQ and HCQ were frequently associated with QT prolongation, heart arrhythmias, development of Torsade des Pointes, and retinopathy. QT prolongation was the most reported AE when treating COVID-19, and retinopathy was the most reported when treating lupus. The FAERS data was analyzed based on three methods: PRR, Chi-square test, and minimal case number filtering; the results of the analysis were then classified using the OAE. Our FAERS study found that HCQ was associated with 63 significant AEs (including 21 cardiovascular AEs) for COVID-19 patients and 120 significant AEs (including 12 cardiovascular AEs) for lupus patients, and different (Figures 3–5). These results supported our hypothesis that the disease being treated would significantly affect the likelihood of certain CQ/HCQ AEs to be manifested and reported. Lastly, we developed an OAE-based ontological model for semantically representing different components involving drug AE generation, and we illustrated our model using an HCQ AE patient example reported in the PubMed literature database. The CQ/HCQ drug AE study provided in this paper illustrates the strengths of our newly proposed methodology.

This methodology does have several limitations that require careful investigation and addressing. First, FAERS does have several known biases, including under-reporting, duplicates, stimulated reporting, and confounding by comorbidities or other drug treatment (FDA, 2005). Our analysis demonstrated some of these limitations, as rheumatoid arthritis, a labeled indication for HCQ, was one of the top AEs for HCQ reported in FAERS. Additionally, by filtering out low FAERS case counts, we may have missed rare AEs. It’s also possible that different dosages and exposure levels between indications could account for some of the AEs identified. Reported concentrations of HCQ vary widely, as concentrations may be affected by age, gender, comorbidities, and other confounding factors (Browning, 2014). Drug concentration levels could be evaluated in future iterations of this methodology. Additionally, this methodology does not take into account underlying population differences in event rates between indications, although this could also be evaluated in future iterations of this methodology. For example, both SLE and COVID-19 are known to be associated with cardiac complications (Kreps et al., 2018; Caforio, 2021; Murk et al., 2021), but the underlying event rates were not taken into account in this analysis. Similarly, age, gender, and other individual characteristics may play a role in AEs experienced, which were ontologically modeled (Figure 6) but not accounted for in the FAERS data analysis. In the future, we can further explore how the ontology modeling of these different characteristics can be applied for practical data standardization, sharing, and analysis of the FAERS AE data related to these characteristics. Finally, data extraction and compilation were performed manually; future iterations of this methodology will incorporate automatic data extraction. Ontology also supports data to be findable, accessible, interoperable, and reusable (Wilkinson et al., 2016; Wang and He, 2021; Xie et al., 2021). Ontology can be used to support automatic and FAIR data extraction and analysis.

## Conclusion

To compare AEs between drugs (or indications) used for treating diseases under various conditions, a methodology was developed to apply ontological and statistical methods to analyze data from different sources including USPIs, EMBASE and PubMed literature resources, and FAERS database. As a use case, the AEs of CQ and HCQ following their usages for different diseases were systematically surveyed, represented, and analyzed. Our USPI study found fewer cardiovascular AEs associated with HCQ compared to CQ. Our EMBASE and FAERS data analysis showed that these two drugs have different AE profiles when they were used to treat different diseases including COVID-19 and lupus. An OAE ontology modeling with its usage on a HCQ AE example study further identified the semantic relations among components related to drug AE investigations. This study demonstrated that ontologies such as OAE are helpful and accessible tools to catalogue and identify AEs associated with drugs, allowing the public to further understand the correlation between various factors and drug AEs.

## Data Availability

The original contributions presented in the study are included in the article/Supplementary Material, further inquiries can be directed to the corresponding author.
